# 术后辅助化疗对Ⅰ期非小细胞肺癌无瘤生存时间的影响

**DOI:** 10.3779/j.issn.1009-3419.2017.07.10

**Published:** 2017-07-20

**Authors:** 胜祖 彭, 晓 李, 云 王, 军 刘

**Affiliations:** 1 100044 北京，北京大学人民医院胸外科 Department of Thoracic Surgery, Peking University People's Hospital, Beijing 100044, China; 2 030013 太原，山西省肿瘤医院胸外科 Department of Thoracic Surgery, Shanxi Provincial Cancer Hospital, Taiyuan 030013, China

**Keywords:** 肺肿瘤, 辅助化疗, 早期, 无瘤生存时间, Lung neoplasms, Adjuvant chemotherapy, Stage Ⅰ, Disease-free survival

## Abstract

**背景与目的:**

手术是早期肺癌的首选治疗方法，但早期肺癌术后的预后仍有很大差异，术后是否应用辅助化疗也有争议。本研究探讨术后辅助化疗在Ⅰ期非小细胞肺癌患者中的作用，尤其是在高危人群中的作用。

**方法:**

选择北京大学人民医院2009年1月-2013年6月接受手术的Ⅰa期、Ⅰb期肺癌患者，分别以是否行术后化疗分为两组，用*Kaplan*-*Meier*法进行生存分析，比较两组术后无瘤生存时间（disease-free survival, DFS）的差异；并按危险因素个数进行评分，分为0分、1分、≥2分三组，比较三组术后DFS的差异；单独比较术后化疗对≥2分的高危组患者的作用。

**结果:**

经过筛选后共有465例患者纳入研究，Ⅰa期284例，Ⅰb期181例。Ⅰa期化疗组和对照组术后DFS并无明显差异（*P*=0.171），但化疗组生存曲线位于对照组下方，Ⅰb期两组术后DFS也无明显差异（*P*=0.630）。危险因素评分后的三组患者DFS有明显差异（*P* < 0.001），危险因素越多，术后DFS越差，可看作是高危患者。但单独分析显示，术后化疗与否对这部分高危患者的DFS并无显著影响（*P*=0.763）。

**结论:**

术后化疗对早期非小细胞肺癌的DFS并无积极作用，即使是对于具有多个高危因素的Ⅰ期非小细胞肺癌患者，术后化疗也许也不适用。

## Abstract (en)

**Background and objective:**

Surgical resection is the best choice for early lung cancer, but the prognosis of early postoperative lung cancer is still very different, whether or not to apply adjuvant chemotherapy is controversial. This study examines the role of postoperative adjuvant chemotherapy in patients with stage Ⅰ non-small cell lung cancer (NSCLC), particularly in high-risk groups.

**Methods:**

Patients with pathologic stage Ⅰa and stage Ⅰb NSCLC who underwent complete (R0) resection between January 2009 and June 2013 were identified from Peking University People's Hospital and classified into two groups based on postoperative chemotherapy or not. *Kaplan*-*Meier* and *Log*-*rank* tests were used to compare disease free survival (DFS). Scored according to the number of risk factors, all patients were divided into three groups. *Kaplan*-*Meier* and *Log*-*rank* tests were used to compare DFS between them. The effect of postoperative chemotherapy on high-risk group was observed individually.

**Results:**

A total of 465 patients including 284 cases of stage Ⅰa and 181 cases of stage Ⅰb were enrolled in this study. For stage Ⅰa there was no significant difference between the chemotherapy group and the control group in DFS (*P*=0.171), but the survival curve of the chemotherapy group was located below the control group. For stage Ⅰb there was no significant difference between the two groups either (*P*=0.630). But there were significant differences on DFS between the three groups according to the number of risk factors (*P* < 0.001). The more risk factors mean the worse DFS. However, independent analyses showed no significant effect of postoperative chemotherapy on DFS in the high-risk group patients (*P*=0.763).

**Conclusion:**

Postoperative chemotherapy does not have a positive effect on DFS in early stage non-small cell lung cancer, and chemotherapy may not be appropriate even for patients with multiple risk factors.

肺癌是我国最常见的恶性肿瘤之一，其死亡率不论在男性或女性患者中均居首位^[1]^。并且随着人们健康体检意识的增强，在高危人群中应用低剂量螺旋计算机断层扫描（computed tomography, CT）筛查显著提高了早期肺癌的检出率。手术切除是早期肺癌的首选治疗方法，然而，早期肺癌术后的预后仍有很大差异。有文献^[2]^指出Ⅰb期肺癌患者术后5年生存率为60%左右，术后2年内转移和复发率也达到38%，预后仍然较差。如何在早期肺癌患者术后进行合理的辅助治疗一直是研究的重点，而该期患者术后是否需要辅助化疗仍存在争议^[3]^。本研究收集北京大学人民医院早期肺癌手术患者，依据2017年第八版国际抗癌联盟（Union for International Cancer Control, UICC）肿瘤-淋巴结-转移（tumor-node-metastasis, TNM）标准重新分期，选择Ⅰ期非小细胞肺癌（non-small cell lung cancer, NSCLC）患者，研究术后化疗对这部分患者无瘤生存时间的影响，为早期肺癌术后辅助治疗方法的选择提供理论依据。

## 1 资料与方法

### 1.1 一般资料

选择2009年1月-2013年6月于北京大学人民医院接受手术的肺癌患者，手术方式为开胸或胸腔镜、病变切除（楔形或肺段、肺叶）同时行淋巴结系统清扫或采样（N1和N2至少各采样一组），依据2017年第八版UICC TNM分期标准，结合术后病理结果、术前颅脑CT、全身骨扫描和腹部B超，从中筛选出符合标准的Ⅰ期NSCLC患者（即T1aN0M0、T1bN0M0、T1cN0M0、T2aN0M0）。同时排除了少见的特殊类型的NSCLC（包括移行细胞、颗粒细胞、淋巴上皮样癌、腺样囊性癌、恶性黑色素细胞瘤各1例）；排除了术前进行过新辅助放化疗的患者。因为原位腺癌（adenocarcinoma *in situ*, AIS）和微浸润腺癌（microinvasive adenocarcinoma, MIA）术后的5年存活率达到100%^[4]^，所以我们的研究也将术后病理诊断为AIS和MIA的患者排除。最终共入选病例465例，其中男性233例，女性232例。

### 1.2 研究方法

所有患者均进行了规律的术后随访，每6个月1次，最后一次随访时间为2016年6月。我们研究的主要内容为无瘤生存时间（disease-free survival, DFS），主要根据患者术后随访的影像学结果或再次病理结果确定肿瘤复发，并计算DFS，即疾病进展或最后一次随访距手术日的时间，以月为单位计算。最短2个月，最长89个月，中位时间44个月。

以是否进行过术后化疗为标准分别将Ⅰa期、Ⅰb期患者分为对照组和化疗组两组，并分别比较Ⅰa期、Ⅰb期两组术后的无瘤生存时间是否有差异。

根据2011版美国国立综合癌症网络（National Comprehensive Cancer Network, NCCN）提出的NSCLC术后的高危因素，即低分化、神经内分泌瘤、侵犯脉管、楔形切除、侵犯脏层胸膜和肿瘤 > 4 cm，为所有患者进行危险因素评分，每包含上述一个因素得1分，分为0分组、1分组和≥2分组，≥2分者为高危组。因为2017年第8版UICC TNM分期标准中肿瘤直径 > 4 cm已进入Ⅱa期，因此我们的危险因素并不包含此项。对三组患者进行生存分析，验证危险因素对术后复发时间的影响。

把高危组患者按术后是否进行化疗再次分为两组，并进行生存分析，比较术后化疗与否对这部分患者复发时间的影响。

### 1.3 统计学方法

所有数据采用SPSS 19.0软件进行统计分析，用*Kaplan*-*Meier*法进行生存分析，*Log*-*rank*检验进行组间生存率的比较，以*P*＜0.05为差异有统计学意义。

## 2 结果

### 2.1 临床特征

最终入选465例患者，其中Ⅰa期284例，Ⅰb期181例。Ⅰa期中有43例（15.1%）接受了术后辅助化疗，而Ⅰb期中有66例（36.5%）接受了术后辅助化疗，化疗方案主要为含铂类的两药联合方案。患者基本资料见表 1。从表中可以看出，不论是Ⅰa期还是Ⅰb期，对照组和化疗组的肿瘤位置、病理类型、手术方式、分化程度以及肿瘤大小均无统计学差异。在Ⅰb期中，对照组和化疗组的年龄段构成有统计学差异，这可能是由于考虑到年龄因素，大于70岁的患者更谨慎地选择了术后化疗而造成的。

**1:** 465例Ⅰ期NSCLC患者临床特征 Clinical characteristics of 465 patients with stage Ⅰ NSCLC

Characteristic		Ⅰa (*n*=284)		*P*	Ⅰb (*n*=181)		*P*
		Observation (*n*=241)	Chemotherapy (*n*=43)		Observation (*n*=115)	Chemotherapy (*n*=66)	
Sex				0.509			0.168
	Male	122 (50.6%)	19 (44.2%)		63 (54.8%)	29 (43.9%)	
	Female	119 (49.4%)	24 (55.8%)		52 (45.2)	37 (56.1%)	
Age (yr)				0.307			0.000
	30-49	31 (12.9%)	5 (11.6%)		12 (10.4%)	16 (24.2%)	
	50-69	146 (60.6%)	16 (72.1%)		50 (43.5%)	40 (60.6%)	
	≥70	64 (26.5%)	35 (16.3%)		53 (46.1%)	10 (15.2%)	
Tumor location				0.052			0.193
	Upper right lobe	103 (42.7%)	15 (34.9%)		32 (27.8%)	23 (34.8%)	
	Middle right lobe	18 (7.5%)	6 (14.0%)		9 (7.8%)	11 (16.7%)	
	Lower right lobe	31 (12.9%)	11 (25.6%)		27 (23.5%)	13 (19.7%)	
	Upper left lobe	52 (21.6%)	9 (20.9%)		25 (21.7%)	13 (19.7%)	
	Lower left lobe	37 (15.4%)	2 (4.7%)		19 (16.5%)	6 (9.1%)	
	Endobronchial	/	/		3 (2.6%)	0 (0.0%)	
Histologic type				0.637			0.877
	Adenosquamous	3 (1.2%)	1 (2.3%)		1 (0.9%)	1 (1.5%)	
	Squamous	23 (9.5%)	6 (14.0%)		22 (19.1%)	11 (16.7%)	
	Adenocarcinoma	204 (84.6%)	33 (76.7%)		86 (74.8%)	49 (74.2%)	
	Neuroendocrine	11 (4.6%)	3 (7.0%)		6 (5.2%)	5 (7.6%)	
Resection range				0.439			0.888
	Wedge or segment	29 (12.0%)	3 (7.0%)		13 (11.3%)	6 (9.1%)	
	Lobectomy	212 (88.0%)	40 (93.0%)		99 (86.1%)	58 (87.9%)	
	Pneumonectomy	/	/		3 (2.6%)	2 (3.0%)	
	or double						
	lobectomy						
Differentiation degree				0.538			0.680
	Unknown	27 (11.2%)	5 (11.6%)		6 (5.2%)	1 (1.5%)	
	High	66 (27.4%)	7 (16.3%)		8 (7.0%)	7 (10.6%)	
	Middle-high	44 (18.3%)	9 (20.9%)		22 (19.1%)	9 (13.6%)	
	Middle	65 (27.0%)	11 (25.6%)		45 (39.1%)	27 (40.9%)	
	Low-middle	21 (8.7%)	5 (11.6%)		22 (19.1%)	14 (21.2%)	
	Low	18 (7.5 %)	6 (14.0%)		12 (10.4%)	8 (12.1%)	
Tumor diameter (cm)				0.056			0.264
	0＜d≤1	87 (36.1%)	8 (18.6%)		20 (17.4%)	5 (7.6%)	
	1＜d≤2	99 (41.1%)	20 (46.5%)		40 (34.8%)	22 (33.3%)	
	2＜d≤3	55 (22.8%)	15 (34.9%)		31 (27.0%)	22 (33.3%)	
	3＜d≤4	/	/		24 (20.9%)	17 (25.8%)	
Visceral pleural invasion				/			0.604
	Negative	/	/		29 (25.2%)	19 (28.8)	
	Positive	/	/		86 (74.8%)	47(71.2)	
Vascular invasion				0.110			0.174
	Negative	239 (99.2%)	41 (95.3%)		111 (96.5%)	60 (90.9%)	
	Positive	2 (0.8%)	2 (4.7%)		4 (3.5%)	6 (9.1%)	
NSCLC: non-small cell lung cancer.							

### 2.2 单因素分析术后辅助化疗对DFS的影响

用*Kaplan*-*Meier*法分别对Ⅰa期、Ⅰb期的两组患者进行生存分析，结果显示Ⅰa期和Ⅰb期的对照组与化疗组术后DFS均并无明显差异（Ⅰa期：*Log*-*rank*/χ^2^=1.877，*P*=0.171；Ⅰb期：*Log*-*rank*/χ^2^=0.232，*P*=0.630）。但从生存曲线看，在Ⅰa期中化疗组位于对照组下方，即其DFS差于未行化疗的患者。见图 1。

**1:**
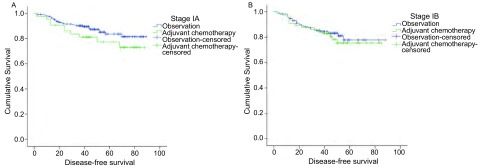
Ⅰ期NSCLC患者术后化疗组和对照组的生存曲线（A：Ⅰa期：*P*=0.171；B：Ⅰb期：*P*=0.630） *Kaplan*-*Meier* survival curves between stage Ⅰ NSCLC patients with chemotherapy and without chemotherapy (A: Stage Ⅰa: *P*=0.171; B: Stage Ⅰb: *P*=0.630)

### 2.3 术后危险因素得分对DFS的影响

按照前文所述的标准对所有研究病例进行危险因素评分后，0分组有231例（49.7%），1分组有176例（37.8%），≥2分组有58例（12.5%）。生存分析结果显示随危险因素得分增加，DFS明显下降，各组比较有明显统计学差异（*Log*-*rank*/χ^2^=26.897, *P*＜0.001），生存曲线见图 2。因此，≥2分组可视为高危组，很有必要研究术后辅助化疗对这部分患者的影响。

**2:**
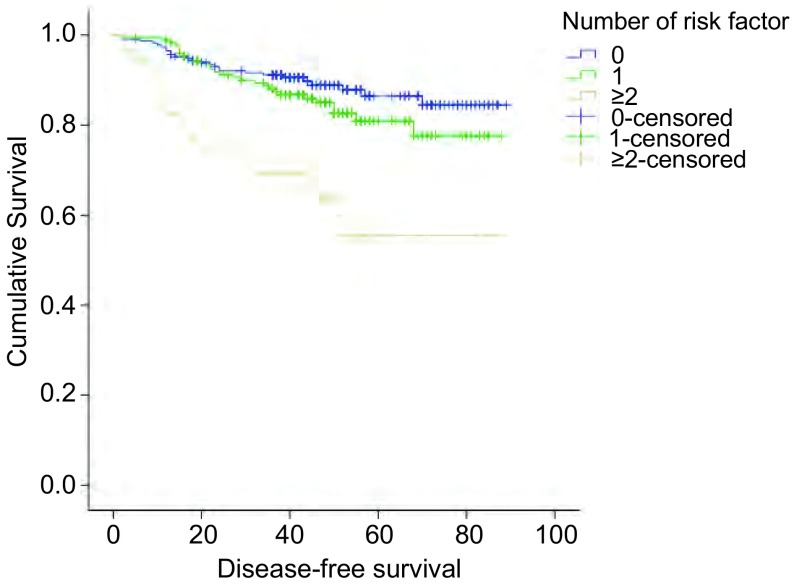
危险因素评分后各组的生存曲线（*P*＜0.001） *Kaplan*-*Meier* survival curves between patients after the risk factor score (*P* < 0.001)

### 2.4 术后化疗对Ⅰ期NSCLC术后高危患者的作用

危险因素得分≥2分的高危患者共58例（Ⅰa期8例，Ⅰb期50例），其中有21例（Ⅰa期1例，Ⅰb期20例）行术后化疗。单独分析术后辅助化疗对这部分患者的作用，结果显示术后化疗与未化疗相比无明显差异（*Log*-*rank*/χ^2^=0.091, *P*=0.763），生存曲线见图 3。

**3:**
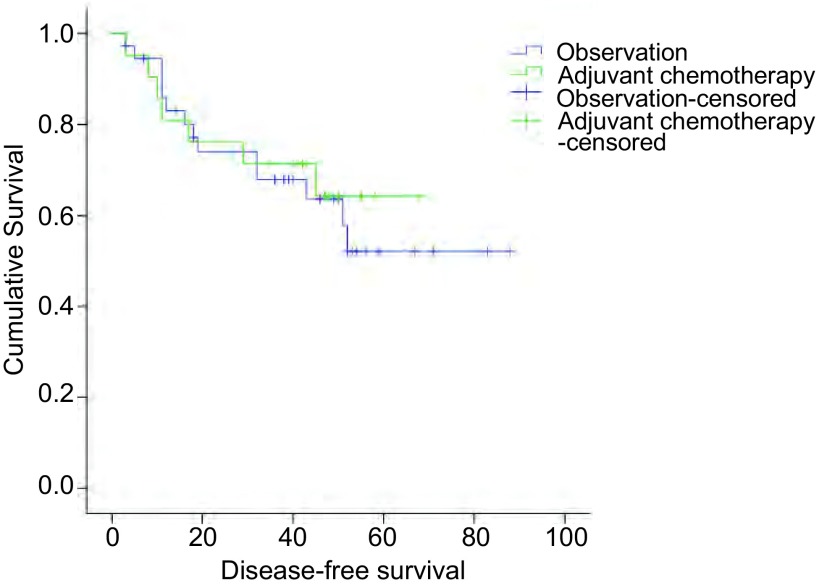
Ⅰ期NSCLC术后高危组患者化疗组和对照组的生存曲线（*P*=0.763） *Kaplan*-*Meier* survival curves between high-risk group patients with chemotherapy and without chemotherapy (*P*=0.763)

## 3 讨论

手术切除是NSCLC的重要治疗手段，但尽管接受了根治性手术治疗，NSCLC患者的5年存活率仍不理想，Ⅰa期为73%，而Ⅲa期仅为24%^[5]^。因此，术后化疗成为NSCLC患者在接受根治性手术治疗后的重要辅助治疗手段。许多研究已经证实了术后化疗在Ⅱ期、Ⅲa期患者的地位，目前主要争议主要集中在术后化疗对Ⅰ期NSCLC患者的作用，尤其是对Ⅰb期患者的作用。在最近的一项包含了34个中心8, 447个病例的回顾性*meta*分析显示，Ⅰb期NSCLC患者接受了以铂类为基础的术后辅助化疗后，将OS从55%提高到60%^[6]^。一项包含了16项研究4, 556例Ⅰb期NSCLC患者的*meta*分析中，术后辅助化也疗显示了明显的OS提高^[7]^。但这种获益仅在接受了尿嘧啶类和替加氟化疗的患者中体现，而接受了四周期铂类为基础的化疗患者中并没有获益。

我们以无瘤生存时间为主要研究终点。这是因为随着治疗手段的不断进步，尤其是靶向治疗的应用，许多出现复发或转移的NSCLC患者接受了更为有效的治疗，存活时间明显延长，所以我们认为以肿瘤进展作为研究终点才能更真实地反映术后辅助化疗的作用。

我们的研究显示术后化疗对Ⅰa期、Ⅰb期NSCLC患者的DFS均无积极作用，化疗组与对照组相比无统计学差异。但从生存曲线上看，Ⅰa期患者术后化疗组位于对照组下方，稍差于对照组。由于基线数据中Ⅰb期对照组和化疗组的年龄段构成有统计学差异，因此我们去除了大于70岁的病例后再次分析，两组年龄段构成则无明显差异（*P*=0.282），再次比较化疗组与对照组的DFS，也得出与之前相似的结果，即仍然无明显差异（*Log*-*rank*/χ^2^=1.555, *P*=0.212）。已经有研究显示，术后化疗对Ⅰa期患者的OS显示了有害的作用，对IB期患者的OS并没有明显提高^[8]^。这与我们的研究结果是相符合的。因此目前的指南不推荐对IA期NSCLC患者进行术后辅助化疗，许多关于Ⅰ期NSCLC患者术后辅助化疗的研究都建议可以考虑对于高危患者进行辅助化疗^[9]^，但究竟效果如何，均没有给出明确答案。

2011版NCCN提出NSCLC术后的高危因素包括低分化、神经内分泌瘤、侵犯脉管、楔形切除、侵犯脏层胸膜和肿瘤 > 4 cm。我们的研究采用2017年第8版UICC TNM标准重新分期，因此已经将肿瘤 > 4 cm的病例排除。从我们入选的病例可以看出，在Ⅰa期的患者中有很多也包含了低分化、神经内分泌瘤、楔形切除和侵犯脉管等危险因素，因此我们根据危险因素评分将所有Ⅰ期患者分为三组。生存分析结果显示，三组间有明显的统计学差异（*P*＜0.001），即包含越多的危险因素则术后DFS越差。因此这些包含2个及更多高危因素的患者似乎更应该接受术后辅助治疗。我们单独研究了术后化疗对于这部分高危患者的作用，然而*Kaplan*-*Meier*分析显示术后化疗与否对DFS并无统计学意义。这一结果提示在目前新的分期标准下，即使是具有多个高危因素的Ⅰ期NSCLC患者，其术后化疗也应慎重选择。不过，按照本研究的评分标准，高危组患者共有58例，数据量偏小，可能对结果有所影响。

本文为回顾性研究，因此这些早期肺癌的患者在术后是否化疗的选择上可能存在偏倚，但是仍可以为将来治疗策略的制订和进一步前瞻性研究的设计提供参考依据。早期NSCLC患者行根治性肺叶切除术后，化疗可能不是首选的辅助治疗手段，也许应该从其他方法上寻求突破，使这部分患者有更好的生存获益。
